# Nanoparticle Vaccines Encompassing the Respiratory Syncytial Virus (RSV) G Protein CX3C Chemokine Motif Induce Robust Immunity Protecting from Challenge and Disease

**DOI:** 10.1371/journal.pone.0074905

**Published:** 2013-09-10

**Authors:** Patricia A. Jorquera, Youngjoo Choi, Katie E. Oakley, Thomas J. Powell, James G. Boyd, Naveen Palath, Lia M. Haynes, Larry J. Anderson, Ralph A. Tripp

**Affiliations:** 1 Department of Infectious Disease, University of Georgia, Athens, Georgia, United States of America; 2 Artificial Cell Technologies, New Haven, Connecticut, United States of America; 3 Division of Viral Diseases, Centers for Disease Control and Prevention, Atlanta, Georgia, United States of America; 4 Department of Pediatrics, Emory University School of Medicine, Atlanta, Georgia, United States of America; University of Iowa, United States of America

## Abstract

Nanoparticle vaccines were produced using layer-by-layer fabrication and incorporating respiratory syncytial virus (RSV) G protein polypeptides comprising the CX3C chemokine motif. BALB/c mice immunized with G protein nanoparticle vaccines produced a neutralizing antibody response that inhibited RSV replication in the lungs following RSV challenge. ELISPOT analysis showed that G nanoparticle vaccinated mice had increased levels of RSV G protein-specific IL-4 and IFN-γ secreting cells compared to controls following RSV challenge. Remarkably, RSV challenge of G protein nanoparticle vaccinated mice resulted in increased RSV M2-specific IL-4 and IFN-γ secreting T cells, and increased M2-specific H-2Kd-tetramer positive CD8^+^ T cells in the lungs compared to controls. Cell type analysis showed vaccination was not associated with increased pulmonary eosinophilia following RSV challenge. These results demonstrate that vaccination of mice with the RSV G protein nanoparticle vaccines induces a potent neutralizing antibody response, increased G protein- and M2- specific T cell responses, and a reduction in RSV disease pathogenesis.

## Introduction

Human respiratory syncytial virus (RSV) is an important viral agent causing serious lower respiratory tract illness in infants, the elderly, and those individuals with cardiopulmonary disease or with impaired immune responses [1–4]. Natural infection with RSV provides incomplete protection from reinfection and disease as demonstrated by the recurrence of even severe RSV infections throughout life [5,6]. Despite decades of effort to develop safe and effective RSV vaccines none have been successful. The first RSV candidate vaccine, a formalin-inactivated alum-precipitated RSV (FI–RSV) preparation did not confer protection and was associated with a greater risk of serious disease with subsequent natural RSV infection [7,8]. Live attenuated and inactivated whole virus vaccine candidates have also failed to protect as they were either insufficiently attenuated or demonstrated the potential for enhanced pulmonary disease upon subsequent RSV infection [9–13]. Subunit vaccines based on the RSV F protein isolated from infected cell culture have been evaluated in adults, children over 12 months of age, and in elderly, but despite being well tolerated the F subunit vaccines were not sufficiently immunogenic [14–19]. Evidence indicates that the RSV F protein is important in inducing protective immunity [16,20], but studies evaluating a BBG2Na vaccine (a fusion protein that consists of the central conserved region of the RSV G protein fused to the albumin binding domain of streptococcal protein G) in combination with different adjuvants and by different routes of administration have shown a role for RSV G protein in protection against RSV [21–23]. Particulate vaccines e.g. virus-like particles (VLPs), nanoparticles and virosomes have been used as new vaccine strategies to potentiate immune response against RSV antigens and have shown promising results [24–30]. A recent study using VLPs demonstrated that mice immunized with VLPs carrying RSV F or G protein had higher viral neutralizing antibodies *in vitro* and significantly decreased lung virus loads *in vivo* after live RSV challenge. However, RSV G protein VLPs showed better protective efficacy than RSV F protein VLPs as determined by the level of virus load in the lungs and morbidity post-challenge [31].

Despite the evidence that RSV G protein can induce protective immunity, G protein has also been implicated in disease pathogenesis [32–35]. One of the disease mechanisms linked to G protein is CX3C chemokine mimicry [36]. RSV G protein has similarities to fractalkine, the only known CX3C chemokine, and has fractalkine-like leukocyte chemotactic activity *in vitro* [36]. RSV G protein acts as a fractalkine receptor antagonist modulating the immune response to infection, and inhibiting fractalkine-mediated responses including altering pulmonary trafficking of CX3CR1^+^ immune cells, and modifying the magnitude and cadence of cytokine and chemokine expression [37,38]. Subunit vaccination with G protein polypeptides spanning the central conserved region of the G protein induces antibodies that block G protein CX3C-CX3CR1 interaction and disease pathogenesis mediated by RSV infection [39]. Mice vaccinated with polypeptides containing the CX3C motif generate antibodies that inhibit G protein CX3C-CX3CR1 binding and chemotaxis, reduce lung virus titers, and prevent body weight loss and pulmonary inflammation [39–41]. Thus, an RSV vaccine that induces antibodies that block G protein CX3C-CX3CR1 interaction should prevent modulation of immune and inflammatory responses to RSV infection.

Particulate vaccines have been shown to induce potent immune responses in the absence of conventional adjuvants due to their recognition by immune cells, as particle structures can simulate natural pathogens such as viruses and bacteria. By incorporating well-defined antigenic epitopes in micro- and nanoparticle constructs, investigators have demonstrated improved immunogenicity of both B and T cell epitopes in a number of model systems including ovalbumin [42], tumor antigens [43,44], hepatitis B antigens [45], RSV antigens [46], and malaria and influenza antigens [47]. The increased potency of nanoparticle vaccine constructs has been attributed to mechanisms including efficient phagocytosis of the particles, cross-presentation, and activation of dendritic cells by increased cytokine production and co-stimulatory marker expression [48–50]. In this study, a novel nanoparticle fabrication method (layer-by-layer deposition; LbL) is used to construct synthetic nanoparticle vaccines that elicit potent humoral and cellular immune responses [42]. LbL nanoparticles produced by the sequential layering of oppositely charged polyelectrolytes on a core substrate were designed to carry one of three designed polypeptides comprising the CX3C motif from the RSV G protein, i.e. GA2, GB1 or GCH17. The results show that mice vaccinated with RSV G nanoparticle vaccines containing the CX3C motif elicit potent antibody responses that neutralize RSV, increase RSV G- and M2-specific T cell responses after RSV challenge, and inhibit pulmonary disease pathogenesis following RSV challenge. These findings suggest that an RSV G protein nanoparticle approach provides a safe and new pathway for producing next-generation RSV vaccines that are effective and prevent RSV G protein-mediated immune modulation and disease pathogenesis.

## Materials and Methods

### Animals

Specific-pathogen-free, 6-to-8 weeks old female BALB/cAnN (H-2^d^) mice (National Cancer Institute, NCI) were used in all experiments. Mice were housed in microisolator cages and were fed sterilized water and food *ad libitum*. All experiments were performed in accordance with the guidelines of the University of Georgia Institutional Animal Care and Use Committee (IACUC), with protocols approved by the University of Georgia IACUC.

### Virus infection

The A2 and B1 strains of RSV were propagated in Vero E6 cells (ATCC CRL-1586) as described [51]. Mice were anesthetized by intraperitoneal administration of Avertin (180-250 mg/kg; Sigma-Aldrich) and intranasally challenged with 10^6^ PFU of RSV A2 in serum-free Dulbecco modified Eagle medium (DMEM; Hyclone, Thermo Scientific).

### Peptide synthesis

Peptides spanning the G protein CX3C motif of the RSV strains A2, CH 17 [52] and B1 were designed for vaccination (Table 1). C-terminal amide peptides were synthesized on a CEM, Liberty^TM^ microwave assisted synthesizer using the manufacturer’s standard synthesis protocols. Crude reduced peptides were partially purified by C_18_ reversed phase HPLC, correct molecular weight was confirmed by electrospray mass spectrometry (ESMS), and then lyophilized. Oxidative refolding was accomplished by dissolving the peptides at 2-5 mg/mL in redox buffer (2.5 mM reduced glutathione, 2.5 mM oxidized glutathione, 100 mM Tris pH 7.0) for 3h at room temperature then at 4°C overnight. Folding was judged complete by a shift to slightly shorter retention time on analytical HPLC. Following a final HPLC purification step refolding was confirmed by a loss of 4.0 (+/- 0.4) amu in the ESMS spectra relative to that of the reduced peptide, as well as an absence of free thiol as detected by DTNB (Ellman’s) assay. Correct disulfide bonding was partially confirmed by ESMS of fragments generated from a thermolysin digest [53] of the synthetic peptide (data not shown). Peptides were aliquoted, lyophilized, and stored at -20°C until use.

**Table 1:** Designed peptides carrying the CX3C chemokine mimic epitope^**a**^ of RSV G protein.

**Name**	**Epitope**	**Peptide sequence**	**RSV strain**
^b^GA2	169-198	NFVP**C**SI**C**S NNPT**C** WAI **C**KRIPNKKPGKKTK_20_Y	A2
^b^GCH17	169-198	NFVP**C**SI**C**S NNPT**C** WDI **C**KRIPSKKPGKKT K_20_Y	CH17
^b^GB1	169-198	NFVP**C**SI**C**GNNQL**C** K S I**C**KTIPSNKPKKKPK_20_Y	B1

^a^ The location of the CX3C motif in the G protein is underlined.

^b^ GenBank sequences used in this study: AAC14901 (RSV A2), NP_056862 (RSV B1) and AF065255 (NY/CH17/93).

### Nanoparticle fabrication and quality control

Nanoparticles were constructed as previously described [42] on 50 nm diameter CaCO_3_ cores by alternately layering poly-l-glutamic acid (PGA, negative charge) and poly-l-lysine (PLL, positive charge) to build up a seven-layer film where the designed peptide (DP) containing the RSV G protein CX3C motif linked to a cationic sequence was added as the outermost layer. The compositions of the films were determined by amino acid analysis (AAA) which showed that comparable amounts of the three peptide components were present in each batch (Table 2). Endotoxin levels were measured using limulus amebocyte lysate (LAL) assay and were found to be less than 0.1 EU/ug of G peptide. The dispersity of the particle vaccines was monitored by dynamic light scattering (DLS). Stepwise LbL steadily increases the diameter of the particles several fold, from an apparent diameter of about 150 nm for uncoated particles to about 400-500 nm for fully coated particles. Some particle aggregation was detected in each batch with a second population of particles in the 1500-2000 nm range.

**Table 2:** Quality control data for nanoparticle vaccines.

**^a^DP name**	**Concentration by ^d^AAA g/ml at 1% solid suspension**			**EndotoxinEU/μg ^a^DP**	**Avg. particle diameter by ^e^DLS nm (number %**)	
	**^a^DP**	**^b^PLL**	**^c^PGA**		**Population 1**	**Population 2**
GA2	57	138	227	0.02	482 (87%)	1590 (13%)
GCH17	47	126	210	0.07	697 (100%)	
GB1	44	127	218	0.05	437 (82%)	1710 (18%)

^a^ DP: designed peptide, ^b^ PLL: poly-l-lysine, ^c^ PGA: poly-l-glutamic acid, ^d^ AAA: amino acid analysis and ^e^ DLS: dynamic light scattering.

### Vaccination

LbL nanoparticles were suspended in phosphate buffered saline (PBS; Hyclone, Thermo Scientific) and dispersed by water bath sonication immediately prior to immunization. Doses were adjusted to deliver either 50 µg DP/100 µL/mouse. Mice were immunized without adjuvant subcutaneously (s.c.) between the shoulder blades on day 0 and boosted on day 21 with the same dose of vaccine used during prime immunization. The control groups received either 10^5^ PFU of live RSV A2 by intranasal instillation (positive control for protection), 100 µL of PBS per injection (negative control) or 10 µg of purified RSV A2 G protein as previously described [36], dissolved in PBS and emulsified with TiterMax® at a 1:1 ratio per injection (positive control for disease enhancement). The antisera from controls and nanoparticle-vaccinated mice were collected at 21 days post boost immunization and stored at -80°C until use.

### Quantification of RSV G and F proteins in virus preparation

Proteins in virus preparations were resolved in polyacrylamide gels as previously described [36]. Silver staining of proteins in the polyacrylamide gels was accomplished as recommended by the manufacturer (Pierce). For quantification of individual proteins in the polyacrylamide gels, different concentrations of bovine serum albumin (BSA) were electrophoresed in the same gel. A standard curve based on the stain of the BSA was used to determine the concentration of the glycoproteins in the virus. For Western analysis, proteins in the polyacrylamide gels were transferred to polyvinylidene difluoride (PVDF) membranes and detected in the blots as previously described [36].

### Indirect ELISA

RSV A2- and B1-specific IgG antibodies were detected by ELISA using 96-well high binding plates (Corning, NY) coated with 10^6^ PFU/mL RSV A2 or B1 in 0.05 M carbonate-bicarbonate buffer, pH 9.6. Sera were added to plates in serial dilutions. RSV-specific antibodies were detected with horseradish peroxidase (HRP) conjugated antibodies specific for mouse IgG, mouse IgG1 or mouse IgG2a (Southern Biotech) followed by addition of SureBlue TMB 1-Component Microwell Peroxidase Substrate (KPL, Inc.) for 15 min. Antibody titers were determined as the last sample dilution that generated an OD_450_ reading of greater than 0.2.

### RSV plaque inhibition assay

Sera obtained from vaccinated and naïve mice were heat-inactivated at 56°C for 30 min, and serial two-fold dilutions starting at a dilution of 1:20 were made in serum-free DMEM. Equal volumes of serum dilutions and RSV A2 previously titrated to yield 200 PFU/200 µL/ well of final mixture were incubated at 37°C and 5% CO_2_ for 1h. Confluent monolayers of Vero E6 cells prepared in 24-well plates were infected with 200 µL/ well, in triplicate, of the serum-virus mixture. After virus adsorption for 2h at 37°C, the cell monolayers were overlaid with 2% methylcellulose media (DMEM, supplemented with 2% fetal bovine serum and 2% methylcellulose). Plates were incubated at 37°C and 5% CO_2_ for 5 days. The cells were then fixed with ice-cold acetone: methanol (60:40) and incubated with a mouse monoclonal antibody specific for RSV F protein (clone 131-2A) followed by a secondary goat anti-mouse IgG antibody conjugated to alkaline phosphatase (AP) (Invitrogen). Plaques were developed using 200 µL/well of 1-Step ^TM^NBT/BCIP (Thermo Scientific) at room temperature for 15 min. Plaques were counted using a dissecting microscope. Titers were calculated from the averages of triplicate sample wells and the reciprocal dilution of the sera completely inhibiting infection was considered as neutralizing antibody titer.

### Lung virus titers

RSV lung virus titers in vaccinated and control mice were determined as previously described [51]. Briefly, lungs were aseptically removed from mice at day 5 post-RSV A2 challenge (10^6^ PFU /mouse), and individual lung specimens were homogenized at 4°C in 1 mL of serum-free DMEM/ high glucose (Hyclone) by use of gentleMACS™ Dissociator (Miltenyi Biotec). Samples were centrifuged for 10 min at 200 xg the supernatants were transferred to a new tube and used immediately or stored at -80°C until they were assayed. For the plaque assay, 10-fold serial dilutions of the lung homogenates were added to 90% confluent Vero E6 cell monolayers. Following adsorption for 1h at 37°C, cell monolayers were overlaid with 2% methylcellulose media and incubated at 37°C for 5 days. The plaques were enumerated by immunostaining with monoclonal antibodies against RSV F protein (clone 131-2A) as described above.

### Histopathological evaluation

Lungs from vaccinated mice were removed 5 days post challenge, perfused with 10% buffered formalin through the heart and trachea and fixed in 10% buffered formalin. The sections were embedded in paraffin, cut in 5 µm-thick sections and stained with hematoxylin and eosin. The sections were evaluated by light microscopy. A histological score for each lung was determined according to the following criteria: 0 = no lung abnormality; 1= <10% of airways inflamed; 2= 10 -30% of airways inflamed; 3= 30 -50% of airways inflamed and 4= >50% of airways inflamed [54]. The slides were evaluated without knowledge of the type of mouse or exposure to antigen. The area covered by an eye piece grid was judged to be normal or abnormal.

### ELISPOT analysis

The day before the assay, 96-well Multiscreen plates (Millipore) were coated with the anti-mouse IL-4 or anti-mouse IFN-γ capture antibody (R&D Systems) and incubated overnight at 4°C. The plates were then blocked by the addition of 200 µL of RPMI-10 media (RPMI 1640 supplemented with 10% FBS, 100 U/mL penicillin, 100 µg/mL streptomycin, 50 µM 2-mercaptoethanol and 2 mM L-glutamine) and incubated for 2 h at 37°C. In parallel, spleens were harvested from vaccinated and naïve mice at 5 days post challenge with RSV A2 and prepared to a single cell suspension using a syringe plunger and a 70 µm mesh nylon strainer. The cell suspensions were collected by centrifugation for 10 min at 200 x g and suspended in RPMI-10 at a concentration of 10^7^ cells/mL. Spleen cell suspensions were added to each well, and cells were stimulated with either 5 µg/mL M2_82-90_ (SYIGSINNI), 5 µg/mL G**_183-197_** (WAICKRIPNKKPGKK) or without peptide for 24h at 37°C and 5% CO_2_. Plates were washed 4 times with wash buffer (0.05% Tween-20 in PBS), anti-mouse IL-4 or anti-mouse IFN-γ detection antibody (R&D Systems) was added and plates were incubated overnight at 4°C. Detection antibody was removed, plates were washed and cytokine spots were developed using ELISpot blue color module (R&D Systems). Spots were counted using an ELISPOT reader (*AID EliSpot Reader* System). RSV-specific ELISPOT numbers were determined from triplicate wells/cell population by subtracting the mean number of ELISPOTs in the unstimulated wells.

### BAL collection and quantification of cytokines

Five days post-challenge, a subset of mice from each group was sacrificed and tracheotomy was performed. The mouse lungs were flushed three times with 1 ml of PBS and the retained BAL was centrifuged at 400 x *g* for 5 min at 4°C. The recovered supernatants were collected and stored at -80°C until assessed for cytokine concentration, and the cell pellet were resuspended in 200 µL of FACS staining buffer (PBS containing 1% BSA). Total cell numbers were counted using a hemocytometer. The Luminex® xMAP™ system using a MILLIPLEX MAP mouse cytokine immunoassay (MCYTOMAG-70K, Millipore) was used to quantitate cytokines in BAL supernatants according to the manufacturer protocol. Briefly, beads coupled with anti-IL-4, anti-IL-5, anti-IFN-γ, anti-IL17A, anti-TNF-α and anti-IL-13 monoclonal antibodies were sonicated, mixed, and diluted 1:50 in assay buffer. For the assay, 25 µL of beads were were mixed with 25 µL of PBS, 25 µL of assay buffer and 25 µL of BAL supernatant and incubated overnight at 4°C. After washing, beads were incubated with biotinylated detection antibodies for 1 h and the reaction mixture was then incubated with streptavidin-phycoerythrin (PE) conjugate for 30 min at room temperature, washed, and resuspended in PBS. The assay was analyzed on a Luminex 200 instrument (Luminex Corporation, Austin, TX) using Luminex xPONENT 3.1 software.

### Flow cytometry

For flow cytometry analysis, cell suspensions were incubated in FACS staining buffer and blocked with FcγIII/II receptor antibody (BD), and subsequently stained with antibodies from BD bioscience, i.e. PE-Cy7 or PE-conjugated anti-CD3e (145-2C11), PerCP-Cy5.5 or FITC -conjugated anti-CD8α (53-6.7), PerCP-Cy5.5-conjugated anti-CD4 (RM4-5) and optimized concentration of APC-conjugated MHC class I H-2K^d^ tetramer complexes bearing the peptide SYIGSINNI (Beckman Coulter) representing the immunodominant epitope of the RSV M2-1 protein [55]. To determine cell types in lungs, cell suspensions were stained for 30 min at 4°C with an optimized concentration of PerCP-Cy5.5-conjugated anti-CD45 (30-F11), FITC-conjugated anti-CD11c (HL3), or PE-conjugated anti-SiglecF (E50-2440). Cells were acquired on a LSRII flow cytometer (BD bioscience) with data analyzed using FlowJo software (v 7.6.5). Based on cell surface markers expression two different cell type were identified: CD45^+^SiglecF^+^ CD11c^low^ as eosinophils and CD45^+^SiglecF ^+^ CD11c^high^ as alveolar macrophages [56].

### Statistics

All statistical analyses were performed using GraphPad software (San Diego, CA). Statistical signiﬁcance was determined using One-way ANOVA followed by Bonferroni’s post-hoc comparisons tests, a *p* value < 0.05 was considered significant.

## Results

### RSV G nanoparticle vaccination induces robust antibody responses

**Figure 1:**
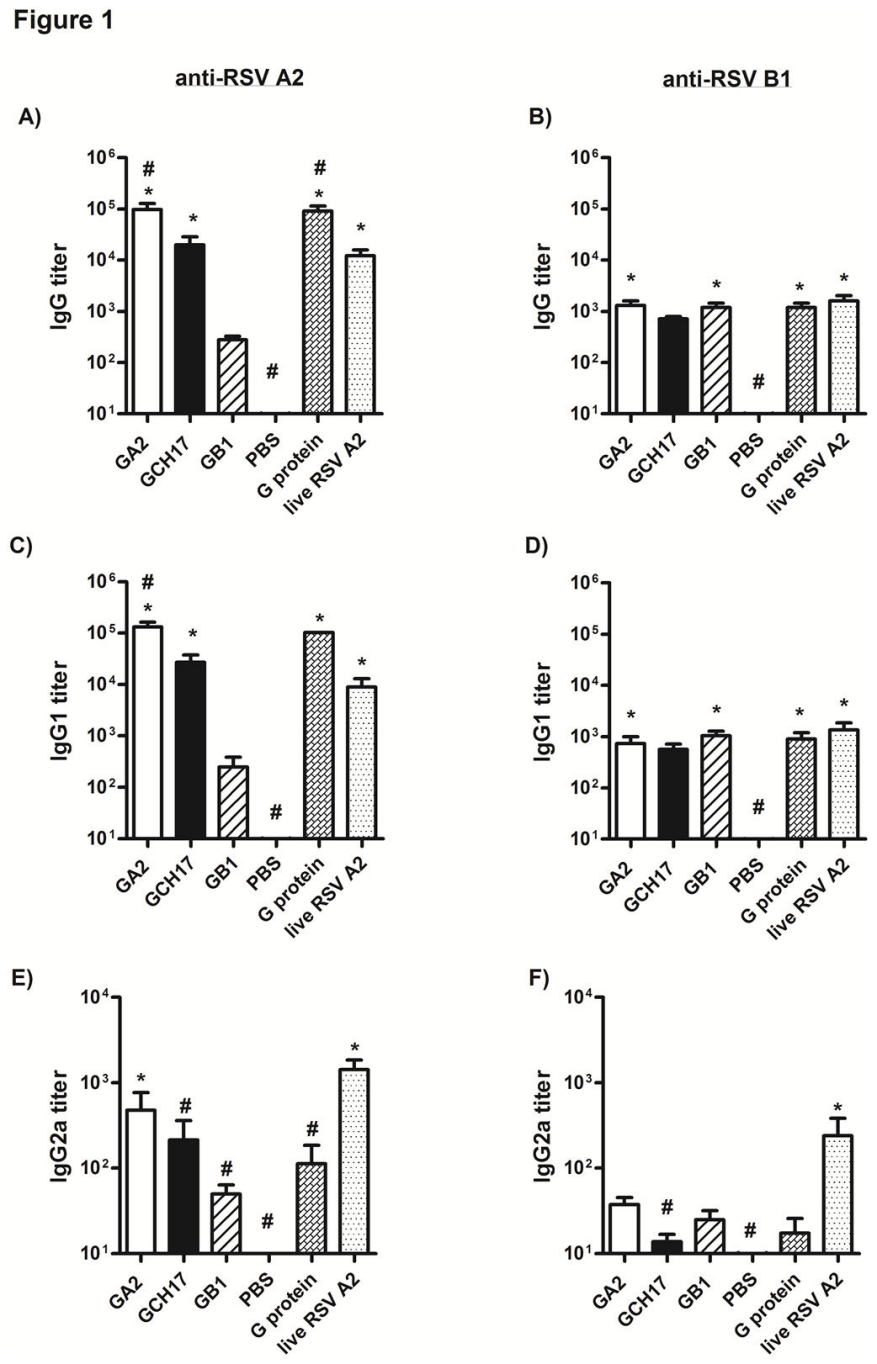
Antibody responses elicited by vaccination with RSV G protein nanoparticles. Groups of BALB/c mice (n=5) were vaccinated with nanoparticle vaccines diluted in PBS to yield 50 µg of designed peptide per dose, with 10 µg of purified G protein emulsified 1:1 with TiterMAX®, 10^5^ PFU of live RSV A2 or with 100 µL of PBS. Sera were obtained from blood taken 21 days after the secondary inoculation. RSV A2 (A, C, and E) and RSV B1 (B, D, and F)-specific IgG (A and B), IgG1 (C and D) and IgG2a (E and F) levels were determined by indirect ELISA. Bars represent the average titer of each group with error bars representing the SEM from n=5 mice per group. *, #, p<0.05, significant difference as determined by one-way ANOVA and Dunnett’s test, compared with PBS vaccinated control mice (*) or compared to live RSV A2 vaccinated mice (#).

Nanoparticle vaccines having a RSV G CX3C motif were prepared as previously described [42] where each construct consisted of seven base layers with the designed peptide (DP) added as the eighth and outermost layer. Three nanoparticle vaccines were generated containing different versions of the CX3C motif: GA2 (CWAIC), GCH17 (CWDIC) and GB1 (CKSIC) (Table 1 and Figure S1). BALB/c mice were subcutaneously immunized with 50 µg of GA2, GCH17 or GB1 nanoparticle vaccines in PBS where the dose was based on the amount of designed peptide (DP) delivered. Control groups were age- and sex-matched BALB/c mice that received live RSV A2, PBS or purified RSV G protein. Twenty one days after the second immunization (boost), mice were bled for determination of antibody titers by ELISA using plates coated with either RSV A2 or RSV B1. Immunization with GA2, GCH17, RSV A2 G protein and live RSV A2 elicited high titers of anti-RSV A2 IgG (Figure 1A and Figure S1) and lower but significant (p<0.05) levels of cross-reactive anti-RSV B1 IgG (Figure 1B). IgG levels induced by GA2 and GCH17 were similar to antibody levels induced by purified G protein combined with adjuvant indicating that nanoparticle vaccination without adjuvant induces equivalent humoral responses. Notably, immunization with the GB1 elicited lower antibody titers that reacted against both homologous (RSV B1, Figure 1B) and heterologous (RSV A2, Figure 1A) virus with equivalent potencies. Although both IgG1 (Figure 1C and 1D) and IgG2a (Figure 1E and 1F) subtypes were generated in response to each of the vaccines tested, only GA2 nanoparticle vaccination induced an IgG2a response similar to live RSV A2 vaccination, and only GB1 induced a balanced IgG1/2a response to RSV A2. Nevertheless, IgG1 was the main subtype produced in mice vaccinated with nanoparticles. These results demonstrate that vaccination with RSV G nanoparticles vaccines in the absence of adjuvant can elicit a strong humoral response comprising both IgG subtypes IgG1 and IgG2a.

### Vaccination with RSV G nanoparticles induces neutralizing antibodies

**Figure 2:**
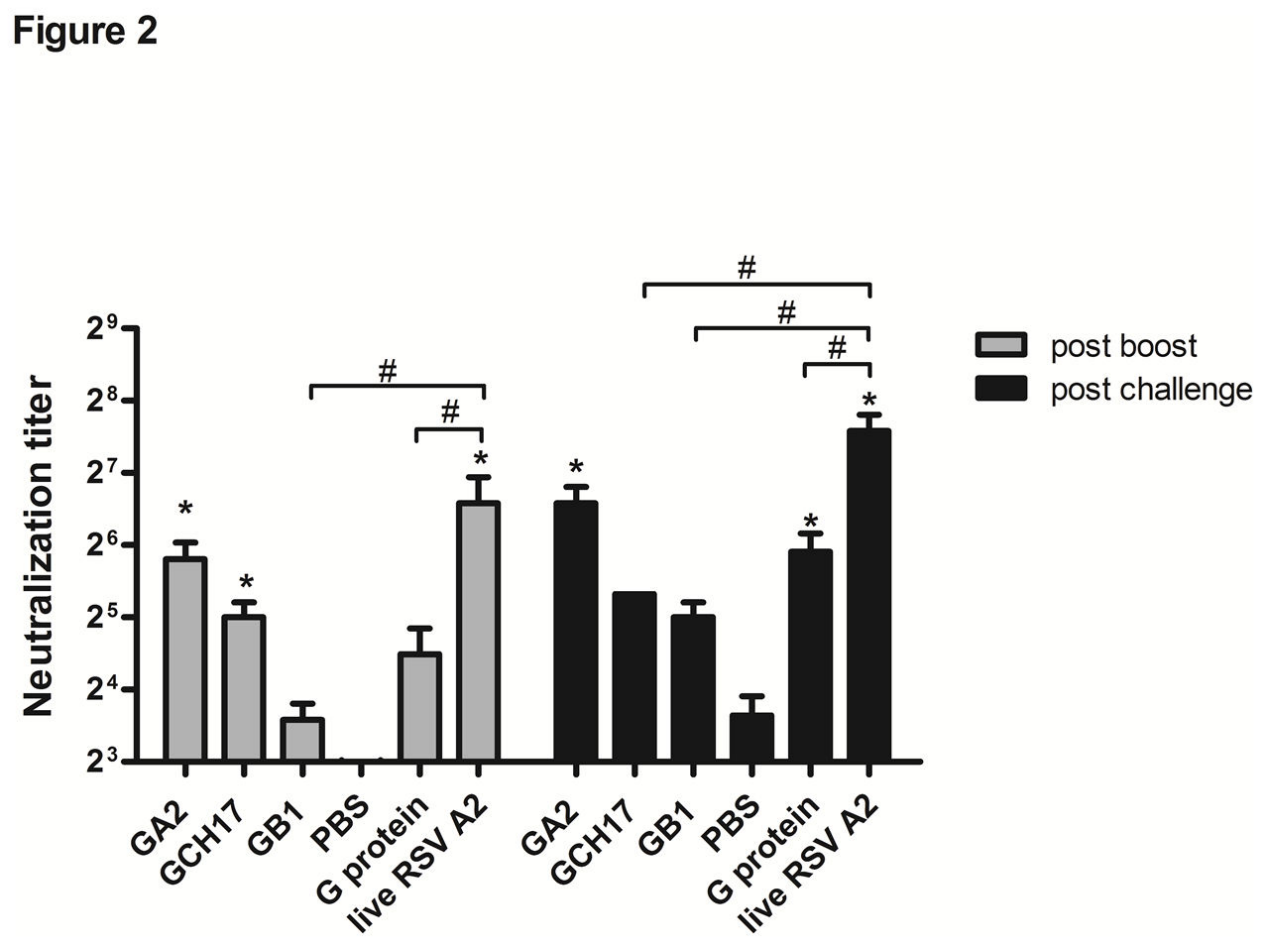
RSV A2 neutralizing antibodies in mice vaccinated with RSV G protein nanoparticles. Sera from vaccinated mice were evaluated for neutralizing antibodies by plaque inhibition assay. Serum samples were collected 21 days after the secondary inoculation (grey bars) and 5 days post challenge (black bars) with 10^6^ PFU of RSV A2. Error bar represents the SEM from n=5 mice per group. *, #, p<0.05, significant difference as determined by one-way ANOVA and Dunnett’s test, compared with PBS vaccinated control mice (*) or compared to live RSV A2 vaccinated mice (#).

Neutralizing antibody is a critical component of an efficacious vaccine. To determine whether vaccination with RSV G protein nanoparticles induced neutralizing antibodies, an *in vitro* plaque inhibition assay was performed using heat-inactivated mouse sera from vaccinated or challenged mice (Figure 2). Three weeks post-boost immunization, and prior to challenge, mice vaccinated with live RSV A2, GA2 nanoparticles or GCH17 nanoparticles showed a significant (p<0.05) increase in neutralizing antibody titer compared to PBS vaccinated mice, while neither GB1 nanoparticles nor G protein vaccinated mice showed substantial increases in neutralization titer compared to the PBS group (Figure 2). Five days post-RSV challenge, the levels of serum neutralizing antibodies increased in all groups, however only GA2 nanoparticle, G protein and live RSV A2 vaccinated mice had antibody titers that were significantly (p<0.05) higher than PBS vaccinated mice (Figure 2). Mice vaccinated with live RSV A2 developed the strongest neutralizing antibody response, nevertheless, there was no significant (p<0.05) difference between live RSV A2 and GA2 nanoparticle vaccinated mice indicating that antibodies specific against the G protein CX3C motif induced by vaccination with the GA2 nanoparticle can block RSV infection *in vitro*.

### RSV G nanoparticle vaccination is protective against challenge

**Figure 3:**
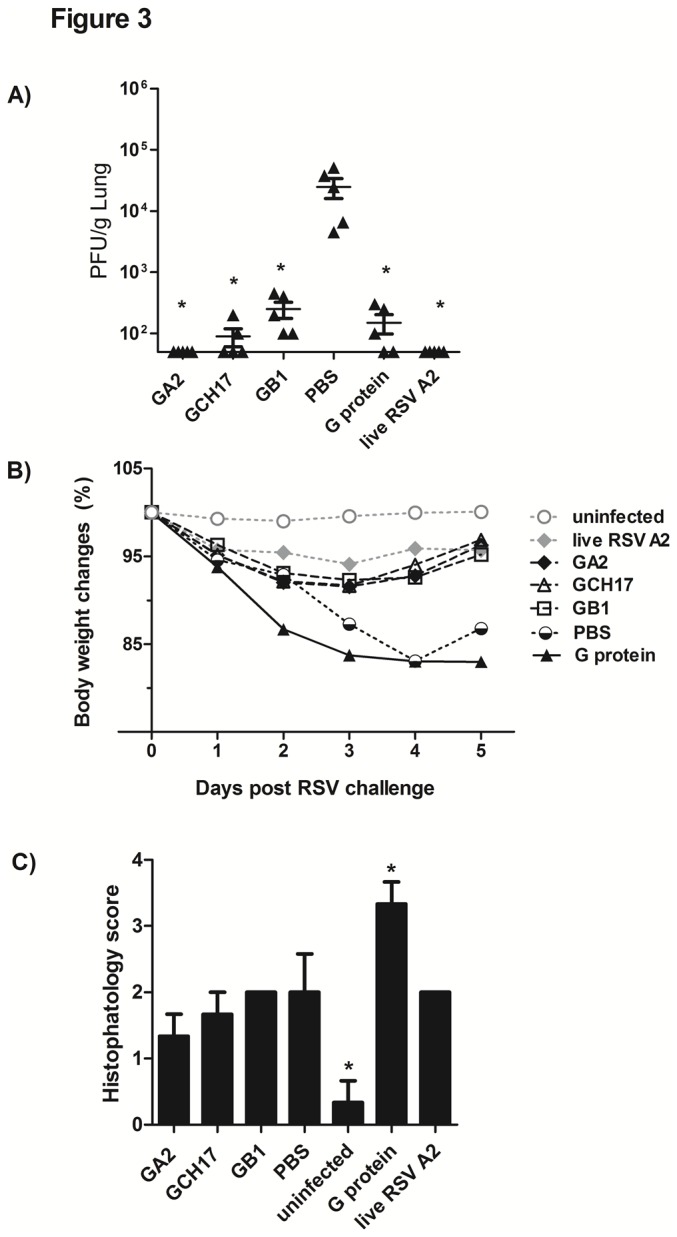
Lung virus titers and body weight loss following RSV A2 challenge of vaccinated mice. Groups of BALB/c mice were vaccinated with nanoparticle diluted in PBS to yield 50 µg of designed peptide per dose, with 10 µg of purified G protein emulsified 1:1 with TiterMAX®, 10^5^ PFU of live RSV A2 or with 100 µL of PBS on days 0 and 21, and challenged i.n. on day 42 with 10^6^ PFU of RSV A2. A) Lung virus titers were determined 5 days post-challenge by plaque assay (n=5). The data are presented as PFU/g of lung tissue. B) Animals were weighed daily and percentage of weight loss calculated based on day 0. C) Quantitation of lung inflammation at day 5 post challenge (n=3). Error bar represents the SEM from n=3-5 mice per group. *, #, p<0.05, significant difference as determined by one-way ANOVA and Dunnett’s test, compared with PBS vaccinated control mice (*) or compared to live RSV A2 vaccinated mice (#).

To evaluate whether immunization with RSV G protein nanoparticles induces protective immunity, vaccinated mice were challenged with RSV A2 virus (10^6^ PFU/mouse) at 6 weeks post-boost vaccination, and the lung virus loads determined at day 5 post-challenge. All vaccinated groups showed a significant (p<0.05) decrease in lung virus loads compared with PBS control, however only mice vaccinated with GA2 nanoparticle and live RSV A2 showed complete inhibition of virus replication (Figure 3A). Importantly, mice immunized with GB1 nanoparticles had reduced lung virus replication indicating vaccination induced a level of cross-protection against RSV A2 challenge. Overall, lung virus titer inversely correlated with the neutralizing antibody titers (Figure 2 and Figure 3A). To determine if vaccination increased disease pathogenesis the percent body weight loss and lung histophatological examination was determined. Mice vaccinated with purified G protein and challenged with RSV A2 immediately lost weight with weight loss peaking at 21% on day 5 post-challenge and showed increased airway inflammation, while mice vaccinated with live RSV, and G nanoparticles developed minor airway inflammation (Figure 3C) and limited weight loss peaking at 5-7% on day 3 post-challenge (Figure 3B). Importantly, G nanoparticle vaccinated mice began to regain lost weight more quickly than the PBS vaccinated mice, suggesting that vaccination with the G protein CX3C motif is associated with improved disease outcome following RSV challenge. These results demonstrate that vaccination with the RSV G nanoparticles induce protective immunity against RSV A2 without inducing increased disease pathogenesis.

### Mice vaccinated with RSV G nanoparticles develop Th1/Th2 memory responses

**Figure 4:**
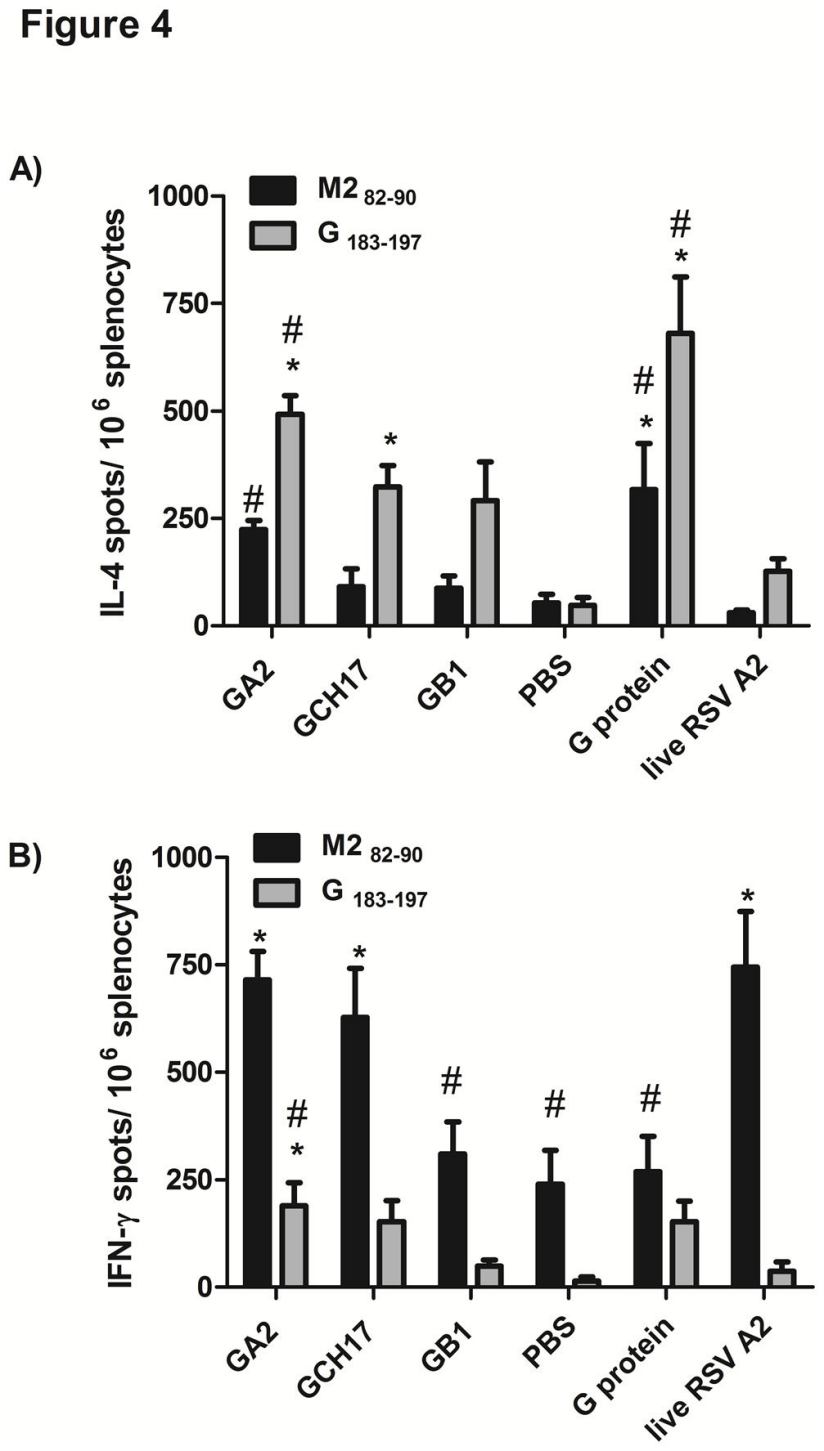
Frequency of RSV-specific IFNγ and IL-4 secreting cells after virus challenge. The number of G_183_-specific (grey bars) and M2_82_-specific (black bars) IL-4 (A) or IFNγ (B) producing splenocytes was determined by ELISPOT in cells harvested 5 days post-challenge. The data are presented as cytokine spots/10^6^ splenocytes. Error bars represent the SEM from n=5 mice per group. *, #, p<0.05, significant difference as determined by one-way ANOVA and Dunnett’s test, compared with PBS vaccinated control mice (*) or compared to live RSV A2 vaccinated mice (#).

Previous studies have reported that vaccinating BALB/c mice with RSV G protein can elicit a Th2-type biased CD4^+^ T cell response upon RSV challenge, and that this skewed Th2-type response is associated with aspects of disease pathogenesis that include airway hyperresponsiveness, mucus over-production, and pulmonary eosinophilia [57–59]. Thus, the outcome of RSV G nanoparticle vaccination and induction of a Th2-type biased T cell response following RSV challenge was investigated. Accordingly, RSV G nanoparticle vaccinated mice were intranasally challenged with RSV A2 at week 6 post-boost and the Th1- (IFN-γ) and Th2-type (IL-4) cell frequencies were measured by ELISPOT assay at day 5 post-challenge. Compared to purified RSV G protein-vaccinated group, mice immunized with GCH17 or GB1 nanoparticle vaccines had lower frequencies of G- and M2-specific IL-4 secreting cells than mice vaccinated with purified G protein, however this difference was not statistically significant (Figure 4A), but similar frequencies of G-specific IFN-γ- secreting cells (Figure 4B). Nevertheless, all vaccinated groups showed higher IL-4 frequencies than the PBS control group (Figure 4A). Remarkably, mice vaccinated GA2 or GCH17 nanoparticle vaccines had a significant (p<0.05) increase in the M2-specific IFN-γ-expressing splenocytes compared to PBS vaccinated mice, and similar to the group vaccinated with live RSV A2 (Figure 4B). Since the nanoparticle vaccines do not have any RSV M2 peptide or related sequences, this finding shows that G nanoparticle vaccination potentiates RSV M2-specific T lymphocyte responses following RSV challenge. Taken together, these results indicate that RSV G nanoparticle vaccination induces strong Th1 and Th2 G protein-specific T cell response.

### Vaccination with RSV G nanoparticles potentiates M2-specific CD8 T cell responses

**Figure 5:**
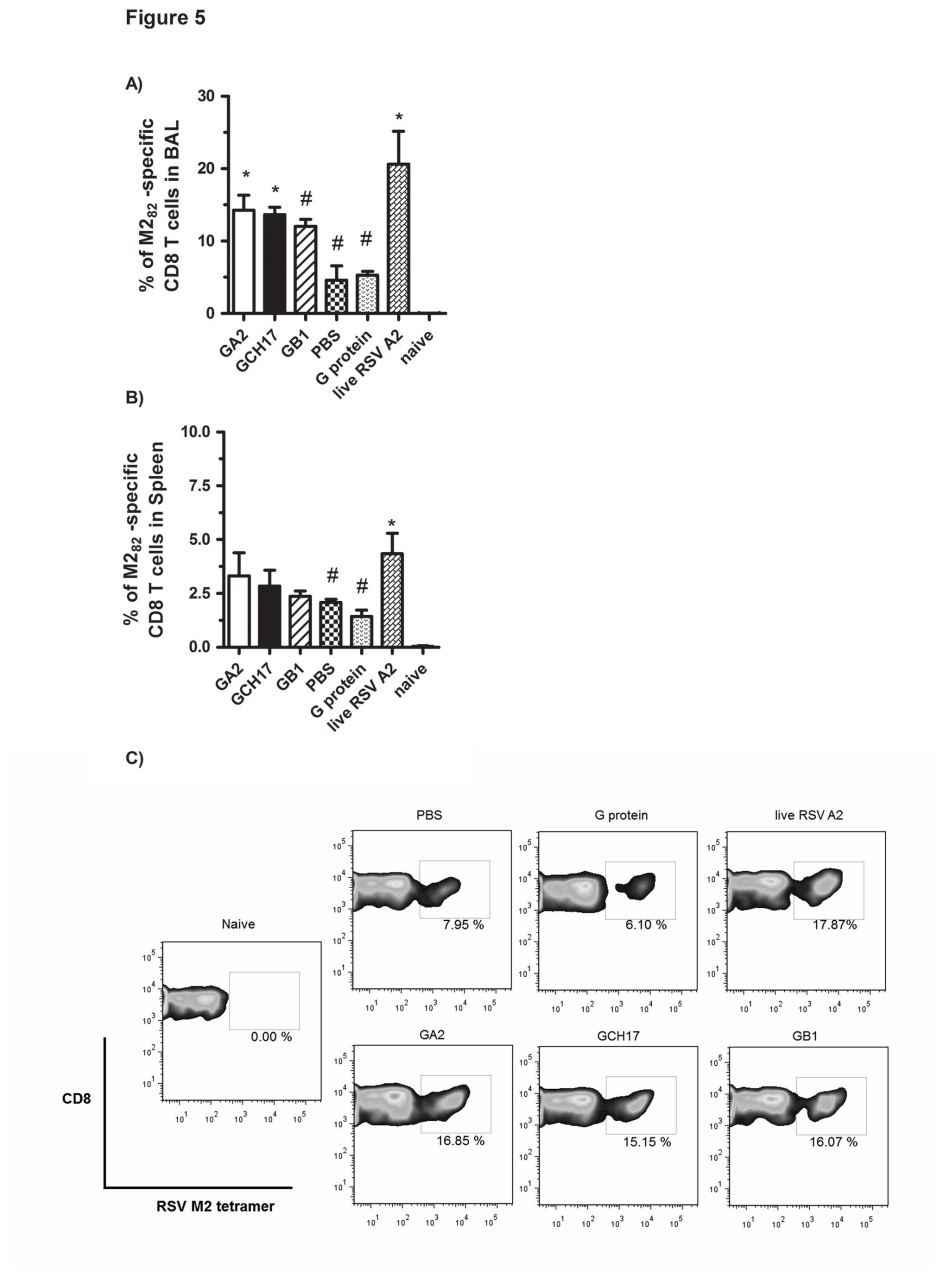
Enumeration of RSV M2-specific CD8^+^ T cells using MHC class I tetramers. Spleen and BAL cell suspensions obtained from mice challenged with RSV A2 were stained with anti-mouse CD3e PE-Cy7-conjugated, anti-mouse CD8α PerCP-conjugated and APC-labeled M2-specific H-2Kd tetramer. FACS contour plots were gate on CD3/CD8 positive cells. Percentage of M2- H-2Kd tetramer positive CD8^+^ T cells in BAL (A and C) and spleen (B) are shown. Error bar represents the SEM from n=5 mice per group. *, #, p<0.05, significant difference as determined by one-way ANOVA and Dunnett’s test, compared with PBS vaccinated control mice (*) or compared to live RSV A2 vaccinated mice (#).

**Figure 6:**
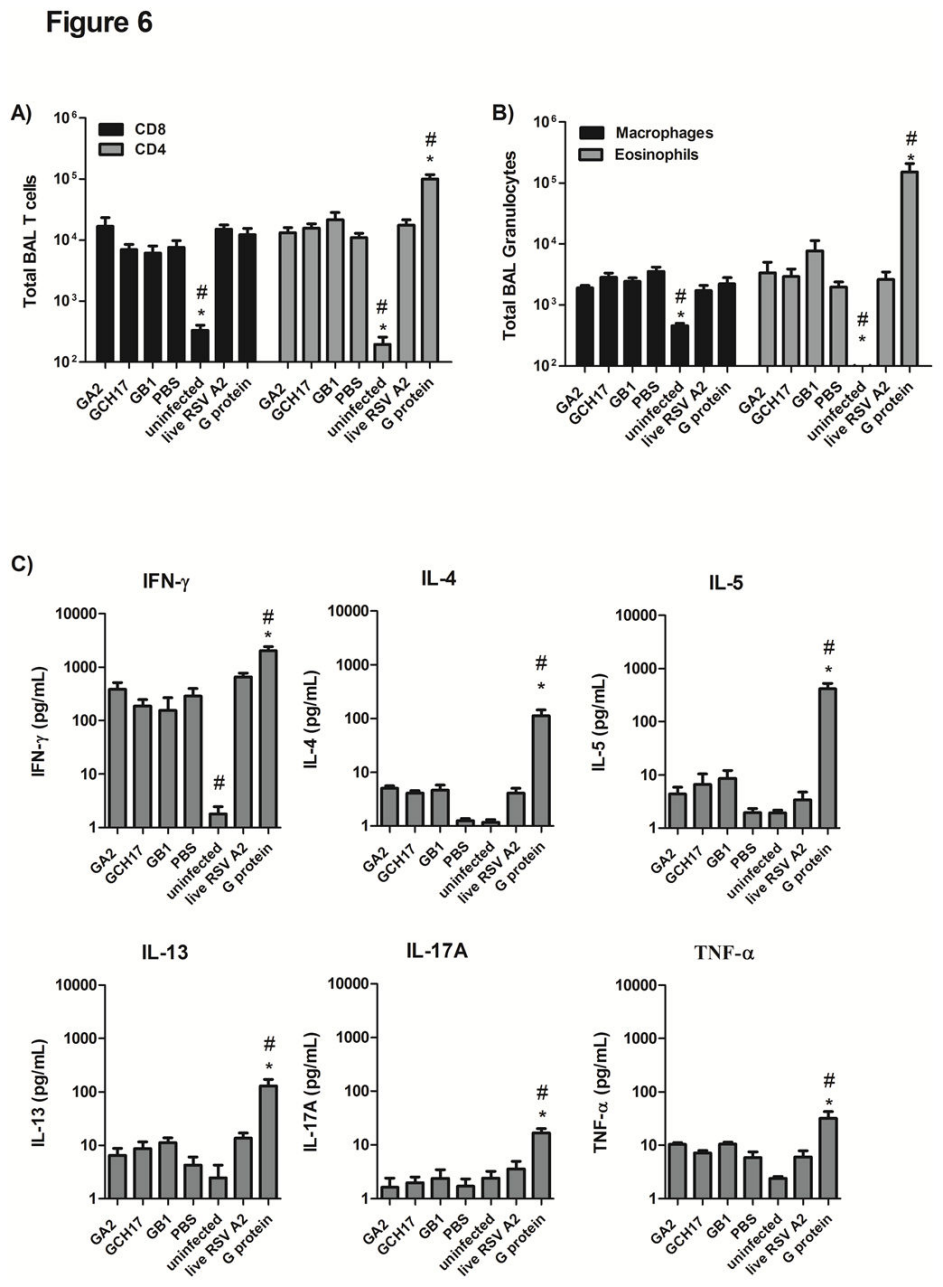
Pulmonary cell recruitment and cytokine production upon RSV infection. Mice were vaccinated with 50 µg of RSV G nanoparticles, 10 µg of purified G protein emulsified 1:1 with TiterMAX®, 10^5^ PFU of live RSV A2 or with 100 µL of PBS and challenged at 6 weeks post boost with 10^6^ PFU of RSV A2. BAL were collected at day 5 post challenge and cell suspensions were immunolabeled with anti-CD3 PE, anti-CD8 FITC and anti-CD4 PerCPCy5.5 to characterize T cells (A) or with a combination of anti-CD45 PerCP-Cy5.5, anti-siglec F PE and anti-CD11c FITC to detect eosinophils (B, grey bars) and macrophages (B, black bars). Based on cell surface markers eosinophils were defined as CD45^+^SiglecF ^+^ CD11c^low^ cells and alveolar macrophages as CD45^+^SiglecF ^+^ CD11c^high^ cells. The data are presented as the total number of cells. Values represent mean ± SEM of cells per BAL (n=5). The level of IFN-γ, IL-4, IL-5, IL-13, IL-17A and TNF-α (C) were measured in BAL supernatant by Luminex assay, and the data are presented as picograms of cytokine/ mL of BAL supernatant (n=5). *, #, p<0.05, significant difference as determined by one-way ANOVA and Dunnett’s test, compared with PBS vaccinated control mice (*) or compared to live RSV A2 vaccinated mice (#).

To address features that may contribute to the increased M2-specific IFNγ-secreting cell frequency in RSV G nanoparticle vaccinated mice challenged with RSV (Figure 4B), spleen and BAL leukocytes from these vaccinated mice were evaluated at day 5 post-RSV A2 challenge, and the number of CD8^+^ T cells positive for RSV M2_82_-_90_ MHC class I H-2K^d^ tetramer determined by flow cytometry. M2-specific CD8^+^ T cells in the spleen were increased following challenge compared to uninfected control mice regardless of prior vaccination (Figure 5B). Upon challenge mice vaccinated with G nanoparticles showed an increase in the percentage of M2-specific CD8^+^ T cells in the BAL that was comparable to vaccinated mice having a recall CD8 T cell response to live RSV A2 (Figure 5A and Figure S2); however, this increase was only statistically significant for the GA2 and GCH17 groups (Figure 5A and 5C). It is important to note that the increase was not linked to an overall increase in the total pulmonary CD8^+^ T cell population (Figure 6A). These results show that vaccination with nanoparticles carrying the central conserve region of the RSV G protein is associated with increased pulmonary recruitment of RSV M2-specific CD8^+^ T cells following RSV challenge. A possible explanation for this outcome is that the immune response against the RSV G CX3C motif induced by vaccination prevents G protein-mediated immune modulation of the CD8^+^ T cell response to RSV infection [37,38] driving a more potent Th1-type and CD8^+^ T cell response to RSV M2 at the site of viral infection.

### Mice vaccinated with RSV G nanoparticles are protected against RSV disease

Previous studies have shown that the magnitude of weight loss correlates with cell recruitment to the lung compartment [32,60,61]. We found that mice vaccinated with G nanoparticles had reduced weight loss and reduced airway inflammation (Figure 3), then to corroborate that vaccination with these nanoparticles was not inducing enhanced disease upon RSV challenge, BAL was evaluated for cell recruitment and cytokines at day 5 post-RSV A2 infection. Mice vaccinated with G nanoparticles had similar numbers of CD4^+^ and CD8^+^ T cells (Figure 6A) in the BAL compared to mice vaccinated with live RSV A2, but lower CD4^+^ T cells numbers than G protein vaccinated mice (Figure 6A). There was no significant increase in pulmonary macrophages in any of the vaccinated groups (Figure 6B). Consistent with previous reports [51,57,58], vaccination with purified G protein induced increased lung eosinophilia upon RSV infection compared to PBS control, or importantly, G nanoparticle vaccinated mice (Figure 6B). Concordantly, analysis of cytokines in BAL fluid showed that the Th1-type cytokine, IFNγ, was increased in all groups upon challenge being significantly (p<0.05) higher in mice vaccinated with G protein (Figure 6C). The Th2-type cytokines IL-4, IL-5 and IL-13 were low in all groups except for mice vaccinated with purified G protein (Figure 6C). Levels of IL-17A and TNF-α were also low in all vaccinated groups except for mice vaccinated with G protein (Figure 6C). Overall, these findings correlate with the histophatological data (Figure 3C) and the granulocyte analysis (Figure 6B) and demonstrate that G protein polypeptide nanoparticle vaccines having the CX3C motif induce a safe and effective immune response similar to that induced by live RSV A2 vaccination, and importantly, protect against RSV disease pathogenesis.

## Discussion

Both humoral and cellular immunity contribute to the host defense against RSV infection [62,63], where neutralizing antibodies are the primary means of protecting against infection, while cell-mediated responses appear to have greater importance for virus clearance [64]. Among RSV antigens, the F protein has been shown to induce neutralizing antibodies and protective immunity in humans and various animal models [65,66]. RSV G has also been shown to induce neutralizing and protective immunity, but to lesser efficiency than F protein [21,67]. However, mixtures of anti-G protein monoclonal antibodies specific for non-overlapping epitopes across the G protein have been shown to be neutralizing due to their synergistic effect, suggesting that polyclonal anti-G protein antibodies can effectively neutralize RSV most likely by steric hindrance that prevents virus binding to the host cell membrane [68]. Importantly, vaccination to induce anti-G protein antibodies that are reactive to the central conserved region of the G protein have been shown to inhibit G protein CX3C-CX3CR1 interaction, to reduce parameters of RSV disease including weight loss, pulmonary inflammation, and lung virus titer [39,40,69,70]. These findings suggest that a RSV G polypeptide vaccination approach to generate antibodies reactive to the central conserved region of the G protein which block G protein CX3C-CX3CR1 interaction may be an effective strategy in developing safe and effective RSV vaccines [40]. Additionally, nanoparticle and particulate vaccines have been shown to induce potent immune responses in the absence of conventional adjuvants due to the recognition by pathogen recognition receptors on a variety of cell types [44,46,48,50], thus combining these strategies offer new vaccine approaches.

In the present study, vaccination with LbL nanoparticle vaccines comprising the conserved RSV G CX3C motif of RSV A2, B1, or CH 17 strains was shown to induce neutralizing antibody responses that inhibit RSV replication upon challenge. Importantly, the findings from this study also show that vaccination with RSV B1- and CH 17-derived G protein nanoparticles induce cross-protection against RSV A2 challenge, a feature important where infection with RSV A strains may mediate more severe disease symptoms compared to RSV B stains [71,72], and because both A and B strains may co-circulate during RSV epidemics [71,72].

Although antibody responses are important for protection against RSV infection, T cell-mediated responses have an important role in virus clearance. In this study, mice vaccinated with RSV G nanoparticles had potent CD8^+^ T cell responses and balanced Th1/Th2 responses to RSV challenge. Previous studies have shown that efficient virus clearance requires Th1-type responses characterized by IFN-γ, IL-2 and IL-12 expression, and that a bias toward Th2-type responses can contribute to RSV pulmonary disease characterized by airway hyperresponsiveness, mucus over-production, wheezing, bronchiolitis, and pulmonary eosinophilia [33,35,73–75]. Accordingly, increased IL-4 expression and polymorphism in the IL-4 gene has been correlated with severe RSV lung disease [76,77] findings consistent with IL-4 mediated differentiation of CD4^+^ T cells toward Th2-type cells [78]. Evidence indicates that CD8^+^ T cells are essential in RSV clearance [79], and that virus clearance is closely associated with an increase of RSV-specific CD8^+^ cytotoxic T cell recruitment and activity in the lungs [80]. Consistent with these findings, this study showed an association between RSV G nanoparticle vaccination-induced cellular responses characterized by increased IFN-γ and IL-4 secreting cells, increased frequencies of M2-specific CD8^+^ T cells, reduced Th2-type cytokines in BAL fluids, and the outcome of reduced RSV replication in the lungs of challenged mice.

Of note, this study showed that mice immunized with nanoparticle vaccines carrying the central conserved region of the RSV G protein had increased percentages of BAL M2-specific CD8^+^ T cells following RSV challenge although none of the G nanoparticle vaccines contained an RSV M2_82-90_ epitope. Consistent with our results, it has been previously reported that in BALB/c mice the RSV G protein, despite lacking H-2^d^-restricted epitopes, is critical and enhances the generation of an effective anti-M2_82−90_ CTL response during RSV infection [81,82]. In addition, Mei and colleagues have reported that CTL responses elicited by vaccines carrying the M2_82−90_ epitope fused to the Measles F protein were greatly enhanced by co-immunization with a recombinant fragment of G containing the conserved central region of the protein [83,84]. The mechanism by which the G protein is enhancing the CTL response to RSV M2 its unknown, however it is possible that vaccination with RSV G nanoparticles induces antibodies that block G protein CX3C-CX3CR1 immune modulatory effects [37,39,40], specifically G protein interference of pulmonary CD8^+^ T cell recruitment in response to RSV infection [37,38]. Accordingly, CX3CR1^+^ CD8^+^ cells are a major component of the cytotoxic response to RSV infection, and that infection with an RSV mutant lacking the G gene dramatically increases the number of CX3CR1^+^ T cells in the lungs and reduces Th2-type cytokine expression [33,75]. Another possible explanation is that RSV G protein-specific memory CD4^+^ T cells induced during the vaccination respond to RSV challenge by expressing Th1-type cytokines/chemokines that contribute to the increased activation and proliferation of CD8^+^ T cells and their subsequent recruitment to the lungs [82]; however, we did not see a significant increase in the production of Th1- type cytokine in BAL fluid of mice vaccinated with G protein nanoparticles. It is also possible that RSV G and M2 proteins may share a common T cell epitope, but this is unlikely since CD4^+^ and CD8^+^ T cells from mice vaccinated with RSV G nanoparticles did not respond to stimulation with M2_82-90_ in the absence of virus challenge (data not shown). Further study is required to characterize and understand the role of anti-G protein immunity on M2-specific CD8^+^ T cell responses.

Overall, the findings from this study show that RSV challenge of BALB/c mice vaccinated with RSV G nanoparticle vaccines is associated with the induction of a neutralizing antibody response, an increase in RSV G- and M2- specific T cell responses, and a reduction in pulmonary disease pathogenesis. Taken together, the findings demonstrate that an LbL G protein nanoparticle vaccination approach for RSV is safe and effective in mice, and offers a new strategy in developing novel RSV vaccines.

## Supporting Information

Figure S1 **Antibody responses elicited by vaccination with RSV G protein nanoparticles and RSV G polypeptides.** Groups of BALB/c mice (n=5) were vaccinated with polypeptides, polypeptides combined with alum, or nanoparticle vaccines diluted in PBS to yield 50 µg of designed polypeptide per dose. RSV A2-specific serum IgG was measured at 21 days after the secondary inoculation. Bars represent the average titer of each group with error bars representing the SEM from n=5 mice per group. *, p<0.05, significant difference as determined by one-way ANOVA and Dunnett’s test.
(TIF)

Figure S2 **Enumeration of RSV M2-specific CD8^+^ T cells using MHC class I tetramers.** BAL cell suspensions obtained from mice challenged with RSV A2 were stained with anti-mouse CD3e PE-Cy7-conjugated, anti-mouse CD8α PerCP-conjugated and APC-labeled M2-specific H-2Kd tetramer. FACS contour plots were gate on CD3/CD8 positive cells. Total numbers of M2- H-2Kd tetramer positive CD8+ T cells are shown. Error bar represents the SEM from n=5 mice per group. *, #, p<0.05, significant difference as determined by one-way ANOVA and Dunnett’s test, compared with PBS vaccinated control mice (*) or compared to live RSV A2 vaccinated mice (#).
(TIF)
