# Supplementary figures and images for: Nanoparticle Vaccines Encompassing the Respiratory Syncytial Virus (RSV) G Protein CX3C Chemokine Motif Induce Robust Immunity Protecting from Challenge and Disease
Source: PLoS One. 2013 Sep 10;8(9):e74905. doi: 10.1371/journal.pone.0074905 (PMC3769300; doi:10.1371/journal.pone.0074905)

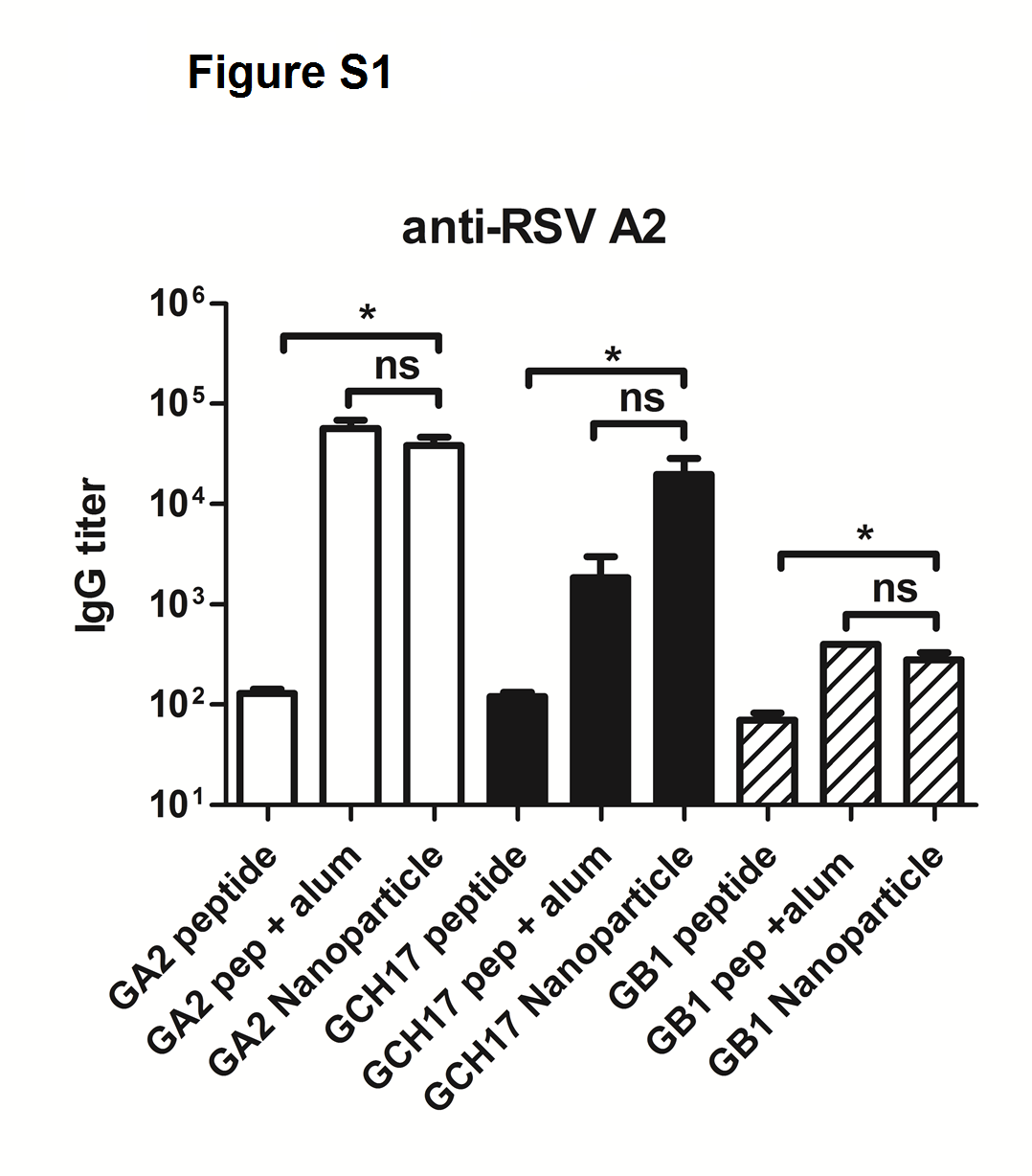

Supplement: Figure S1 — Antibody responses elicited by vaccination with RSV G protein nanoparticles and RSV G polypeptides. Groups of BALB/c mice (n=5) were vaccinated with polypeptides, polypeptides combined with alum, or nanoparticle vaccines diluted in PBS to yield 50 µg of designed polypeptide per dose. RSV A2-specific serum IgG was measured at 21 days after the secondary inoculation. Bars represent the average titer of each group with error bars representing the SEM from n=5 mice per group. *, p<0.05, significant difference as determined by one-way ANOVA and Dunnett’s test. (TIF) [file pone.0074905.s001.tif]

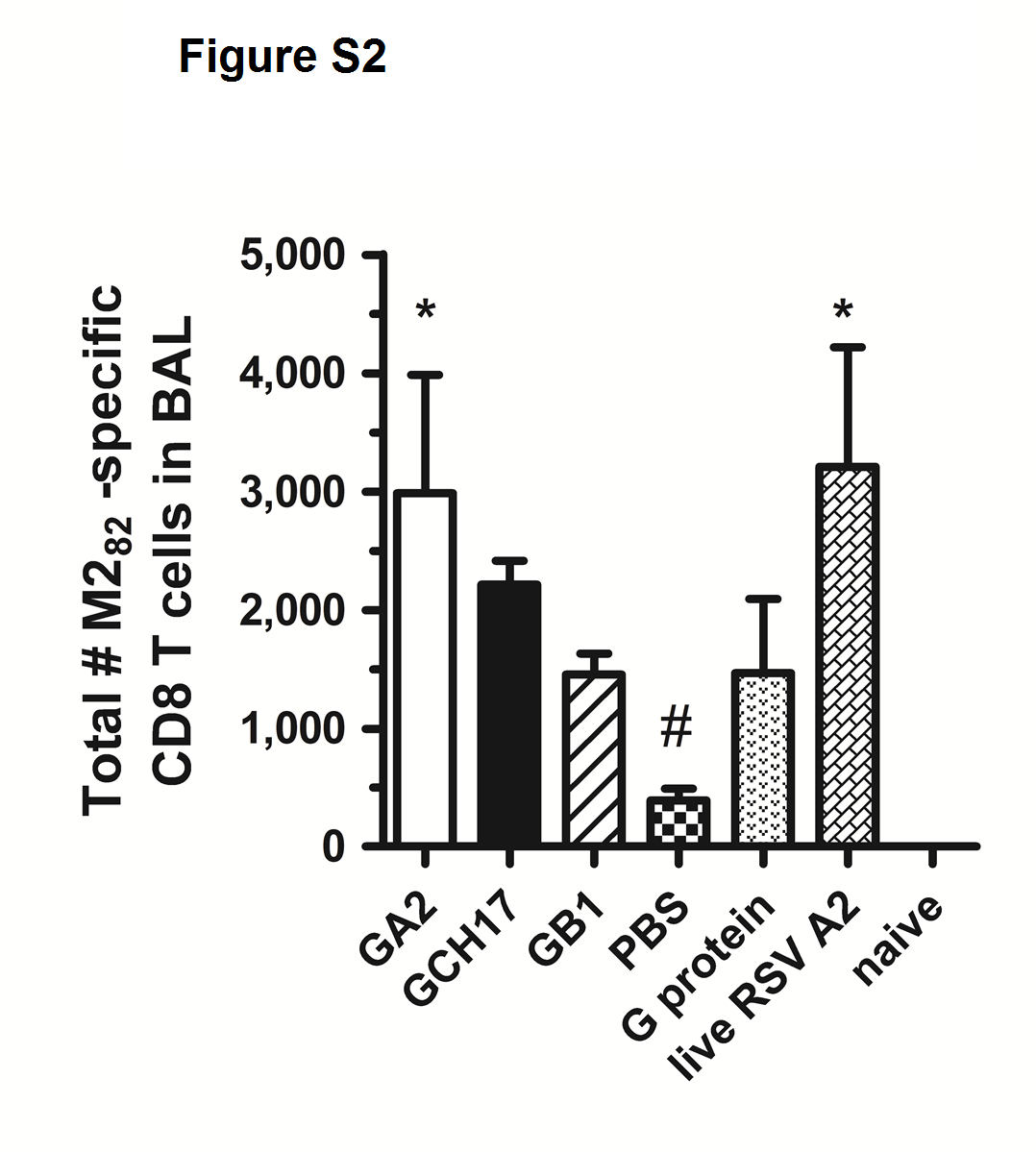

Supplement: Figure S2 — Enumeration of RSV M2-specific CD8+ T cells using MHC class I tetramers. BAL cell suspensions obtained from mice challenged with RSV A2 were stained with anti-mouse CD3e PE-Cy7-conjugated, anti-mouse CD8α PerCP-conjugated and APC-labeled M2-specific H-2Kd tetramer. FACS contour plots were gate on CD3/CD8 positive cells. Total numbers of M2- H-2Kd tetramer positive CD8+ T cells are shown. Error bar represents the SEM from n=5 mice per group. *, #, p<0.05, significant difference as determined by one-way ANOVA and Dunnett’s test, compared with PBS vaccinated control mice (*) or compared to live RSV A2 vaccinated mice (#). (TIF) [file pone.0074905.s002.tif]
